# Incidence, survival and determinants of mortality following mesenteric angiography and continuous intra-arterial prostaglandin E1 perfusion for non-occlusive mesenterial ischaemia in cardiac surgery

**DOI:** 10.1186/1749-8090-8-S1-P87

**Published:** 2013-09-11

**Authors:** T Szerafin, A Horváth, T Maros, I Szentkirályi, L Palotás, P Csizmadia, T Debteceni

**Affiliations:** 1 Department of Cardiac Surgery, Institute of Cardiology of the Medical- and Health Science Centre of the University of Debrecen, Hungary

## Background

Non-occlusive mesenteric ischemia (NOMI) is a rare but severe complication. Early recognition of NOMI is difficult due to its unspecific symptoms and the lack of specific laboratory parameters. Prevention, diagnosis and optimal therapy are under examinations.

## Methods

Our aim was to evaluate the incidence of NOMI, to analyze results of treatment and to identify variables associated with mortality. Hospital records and clinical data of patients treated for NOMI during the last 12 years were reviewed. Clinical outcomes and factors influencing mortality were studied. Statistical analysis was performed to determine the significance of risk factors on mortality with Fisher exact test or χ2 test in categorical variables. Wilcoxon rank test was used to test the continuous variables.

## Results

Between 7/2001and 6/2013 NOMI developed in 64 cases (0.5%). Mean age of patients was 67.9±6.9 year. First symptoms occurred 2.7±2 days after the operation. Mesenteric artery angiography was performed in 59 patients (92.8%). After confirmation of mesenteric vasospasm, a continuous infusion of 2.4 µg/h of prostagalandin E1(PGE1) was administered in the superior mesenteric artery. The 30-day mortality of NOMI after PGE1 treatment was 66. Logistic regression analysis of risk factors revealed preoperative NYHA stadium (P<0.02), presence of internal carotid artery stenosis (P<0.05), prolonged CPB and aortic cross clamp time P<0.01), postoperative arrhythmias (P<0.05), reoperation (P<0.03), use of arterenol and/or IABP ( P<0.05), serum lactate level (P<0.01), length of ventilation (P<0.001 and number of transfused red blood cell units (P<0.05) were independent predictors of mortality.

## Conclusion

Mesenteric ischaemia in cardiac surgery remains a devastating complication. A high index of suspicion with prompt diagnostic evaluation may reduce the delay prior to intervention. Early mesenteric angiography and intraarterial PGE1 infusion seems to be useful to improve survival.

